# Acetabular Aneurysmal Bone Cyst During the Syrian Conflict: A Case Report of Surgical Treatment and Outcomes

**DOI:** 10.7759/cureus.56474

**Published:** 2024-03-19

**Authors:** Mohamad Khatib, Ibrahim W Hasani

**Affiliations:** 1 Surgery, Idlib University Hospital, Idlib, SYR; 2 Biochemistry, Idlib University Hospital, Idlib, SYR; 3 Biochemistry, Mary Private University (MPU), Idlib, SYR; 4 Biochemistry, Al-Shamal Private University (SPU), Idlib, SYR

**Keywords:** chatgpt, syrian conflict, acetabulum, benign bone lesions, aneurysmal bone cysts

## Abstract

Aneurysmal bone cysts (ABCs) are uncommon benign bone lesions that consist of blood-filled vascular spaces surrounded by fibrous tissue septa. Their diagnosis and surgical management are challenging in a war-torn region. In this case report, we present a rare case of a giant aneurysmal bone cyst located around the acetabulum in a 10-year-old female child who presented with an antalgic limp and left hip pain. The lesion was successfully treated with curettage and mixed autologous and synthetic bone grafts, and the follow-up for two years revealed a complete resolution of symptoms and radiological evidence of bone regeneration. This case highlights the successful surgical treatment of a challenging case of ABC in a difficult setting during the Syrian conflict.

## Introduction

Aneurysmal bone cysts (ABCs) are rare benign lesions of bone that typically affect the metaphyseal region of long bones in 60% of cases and 15% in the vertebral column [1,2]. Most cases occur in the lower and upper extremities [3], with 8-12% occurring in the pelvis. The metaphysis is the most common location in long bones, and when the lesion affects the pelvis, it is located in the posterior elements. These non-neoplastic but locally aggressive tumors can cause rapid growth and destruction of the bone, with occasional soft tissue invasion [4]. Regardless of gender, ABCs typically affect young people under the age of 20 [1]. ABCs may be classified into three types: vascular, solid, and a third type, or mixed variant, which represents features of both the vascular and solid types [4,5].

The Syrian conflict, which began in 2011, has resulted in the widespread destruction of infrastructure and the displacement of millions of people. Healthcare in Syria has been severely impacted, with limited access to medical supplies, equipment, and skilled healthcare professionals. As a result, the management of rare medical conditions, such as ABCs, has become even more challenging [6].

The hip joint serves as the pivotal connection between the trunk and the lower limb, serving a crucial role in facilitating movement and force transmission during everyday tasks and physical endeavors, whether in daily routines or athletic pursuits. Notably, its remarkable bony stability inherently influences its biomechanical functions, with variations in osseous anatomy contributing to distinct biomechanical properties unique to each individual's hip joint [7].

Here, we report a rare case of a giant aneurysmal bone cyst located around the acetabulum in a 10-year-old female child who presented with an antalgic limp and left hip pain during the Syrian conflict. The patient underwent surgical treatment with curettage and mixed autologous and synthetic bone grafts, with successful clinical and radiographic outcomes. We highlight the challenges of managing rare medical conditions in the context of the Syrian conflict and discuss the importance of timely and appropriate medical interventions for patients with such conditions. The case is presented with a review of the current literature. Aneurysmal bone cysts are rare, benign lesions of bone tissue. They are composed of vascular spaces that are blood-filled and surrounded by fibrous tissue septa. They are considered pseudocysts because of their lack of epithelial lining.

## Case presentation

A 10-year-old female child presented with a one-year history of a limp and dull pain in the left hip that gradually increased over time. The patient experienced worsening pain at night and with activity but did not display any additional symptoms, such as fevers, chills, night sweats, or weight loss. Prior to referral to an orthopedic surgeon, the patient was unsuccessfully treated with painkillers and non-steroidal anti-inflammatory drugs (NSAIDs) to manage the pain and limp.

**Figure 1:**
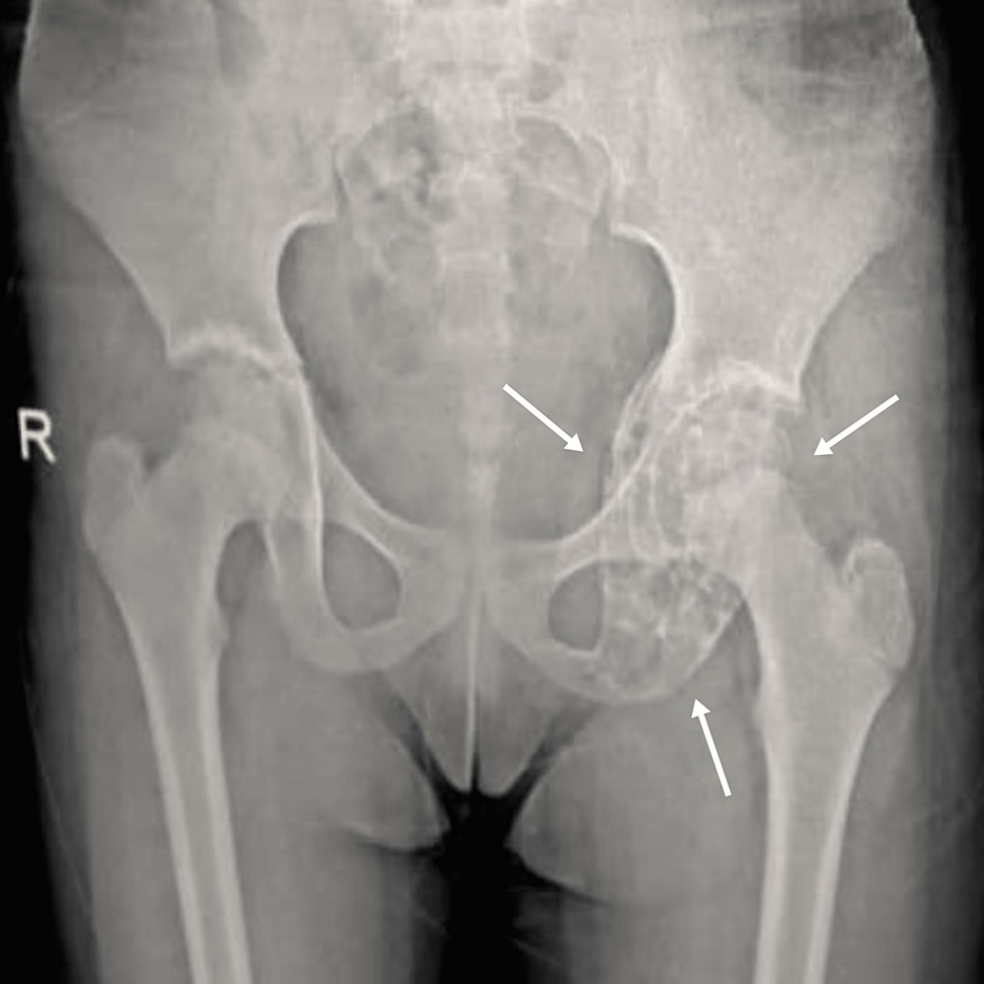
Pre-surgical anteroposterior pelvic and hip X-ray On the pre-operative anteroposterior pelvic X-ray, arrows indicate a periacetabular lobulated lesion that invades the roof of the acetabular cavity and extends below the ischium.

Upon referral, an anteroposterior X-ray of the pelvis and hips revealed a large multilocular radiolucency in the acetabular dome, extending to the ischium and pubic ramus (Figure 1). The laboratory results revealed within-normal-range values, except for anemia and leukocytosis in the cell count, with hemoglobin and white blood cell counts measuring 10.1 g/dL and 12 x 10^9/L, respectively. Additionally, C-reactive protein (CRP) was recorded at 6.2 mg/dL and erythrocyte sedimentation rate (ESR) at 12 mm/hr.

**Figure 2:**
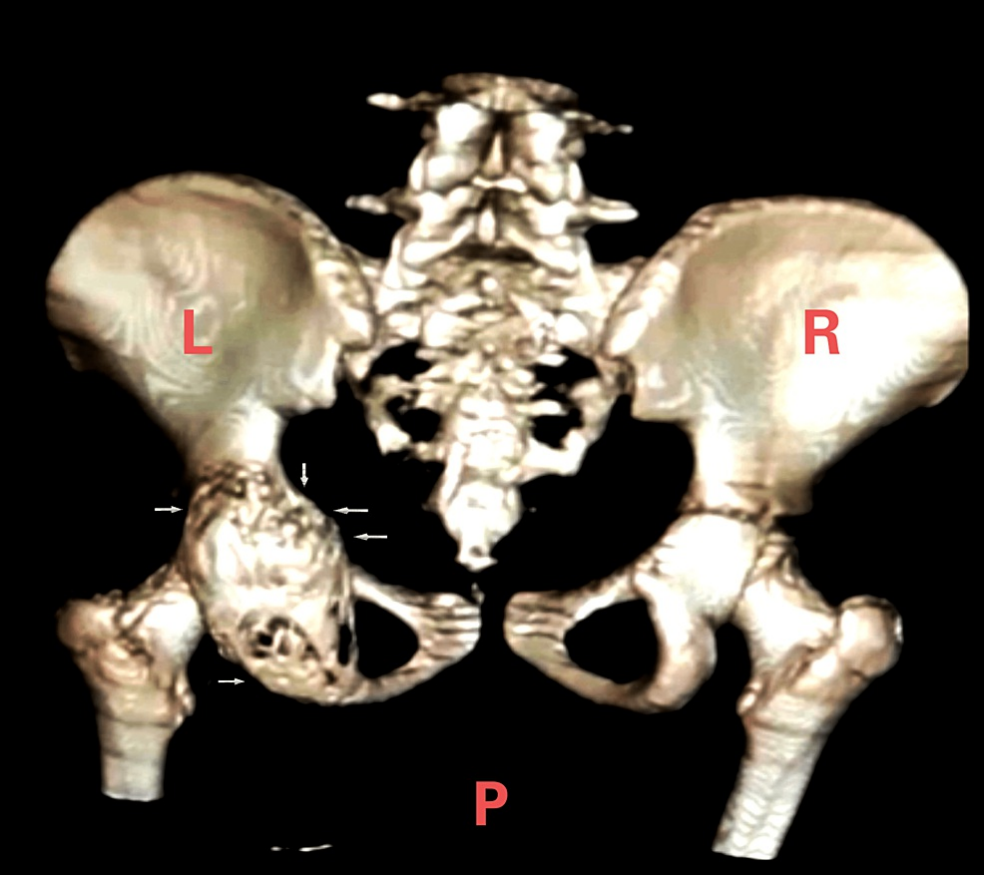
Displays the lesion that was observed via computerized tomography On the computerized tomography, the lesion appeared with an arrow indicating its invasion into the posterior column of the acetabular cavity towards the ischium and extending anteriorly through the floor of the acetabular cavity to the anterior column. L: left; R: right; P: posterior

During the physical examination, restricted hip movements were noted: hip flexion 0-90 degrees, extension 0-5 degrees, abduction 0-20 degrees, external rotation 0-30 degrees, internal rotation 0-20 degrees, along with a palpable lump in the posterior aspect of the gluteal region. A computerized tomography (CT) scan was completed and confirmed the presence of a large lobulated expansile mass with central necrotic hemorrhagic areas, measuring 10 cm in superoinferior dimension and about 7 cm in lateromedial dimension, with a very thin posterior wall. The mass was found to extend partially to the dome of the acetabulum and into the ischium (Figure 2).

Six months prior to surgical intervention, the lesion had been injected with bone marrow from the patient's posterior iliac crest by another surgeon group (first intervention), which did not show clinical or radiographic improvement on control X-rays after five months. In contrast to standard practices, where a biopsy is typically performed if a lesion is detected on imaging studies (X-ray or CT scan) to confirm its nature before any intervention, the circumstances in the patient's case were unique. The individual was admitted to the hospital during a war, and the geopolitical situation prevented the feasibility of conducting a biopsy in the war-torn area. Given these constraints, the option of initially attempting treatment was discussed with the family, considering the potential risks associated with the lack of a confirmed diagnosis.

It is pertinent to note that the patient’s family subsequently sought treatment from another surgeon at Idlib University Hospital, where the situation allowed for a more comprehensive evaluation.

The patient was admitted to the orthopedic ward of Idlib University Hospital, where an incisional biopsy revealed an ABC of the posterior column of the acetabulum extending to the acetabular dome and ischium and the medial wall of the acetabulum.

**Figure 3:**
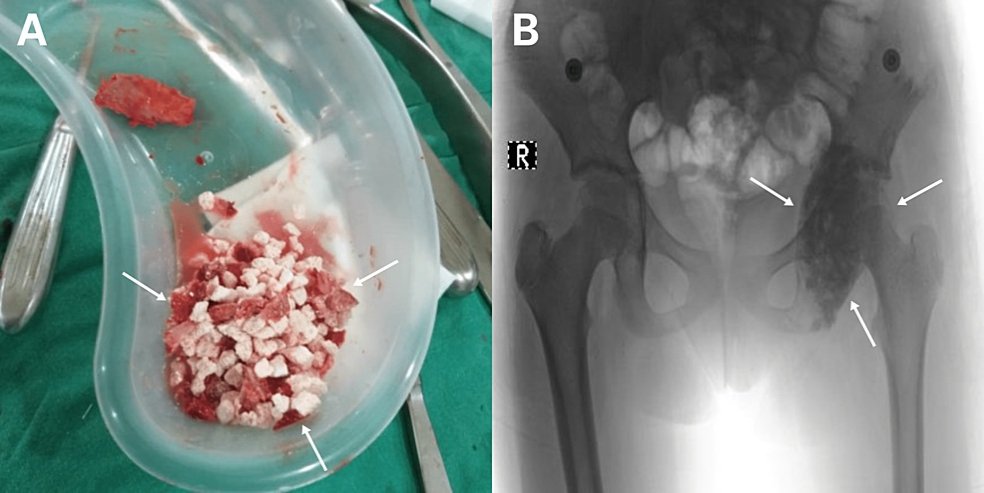
Shows a mixed auto and synthetic graft and the post-surgical X-ray Figure A shows a mixed graft composed of both autograft and synthetic material. In the postoperative X-ray image (Figure B), the mixed graft is visible as it fills the cyst completely following the curettage procedure, as indicated by the arrows.

Following the confirmation of ABC involvement through biopsy, the treatment plan involved complete lesion curettage and the application of mixed auto and synthetic grafts via a posterior approach. The treatment plan involved curettage of the entire lesion and mixed auto and synthetic grafts through a posterior approach (Figure 3), with a vascular surgeon on standby in case of unexpected bleeding that required ligation of the internal iliac artery. Curettage of the entire lesion was performed through a bone window on the posterior column of the acetabulum, followed by mixing the iliac autograft with a synthetic graft to fill the space without requiring intervention by a vascular surgeon.

The acetabular cavity's medial wall was thin, and only standard bone shovels were on hand for scraping. Despite careful scraping of the medial wall, there was a minor penetration of the acetabular cavity's articular surface. This made it impossible to apply 5% phenol before proceeding with the mixed bone graft, as we wanted to prevent phenol from leaking into the hip joint and causing chemical arthritis.

Following the procedure, the patient experienced slight sciatica and numbness in the leg and foot caused by irritation of the sciatic nerve during the operation. However, this issue was resolved before the patient's discharge from the hospital. Subsequently, the patient refrained from bearing weight for six weeks, after which partial weight bearing was permitted. The patient then underwent a physical therapy program aimed at strengthening the pelvic girdle muscles, particularly the gluteal muscles.

Result

After the surgery, the patient's progress was carefully monitored. She was instructed to avoid weight-bearing for six weeks, and pelvic X-rays were taken at each visit to the orthopedic clinic. Three months after the operation, the patient was able to walk without experiencing any pain. A physical examination conducted six months after the operation showed that the patient had a full hip range of motion, including hip flexion of 0-150 degrees, an extension of 0-20 degrees, abduction of 0-45 degrees, external rotation of 0-40 degrees, and internal rotation of 0-45 degrees.

**Figure 4:**
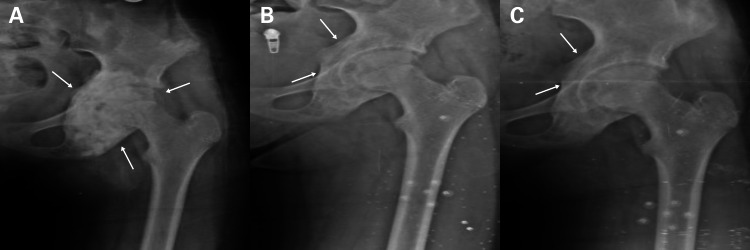
X-ray control after three (A), six (B), and 12 (C) months post-surgery The X-ray follow-up at three, six, and 12 months (A, B, and C) respectively demonstrated satisfactory incorporation of the mixed auto- and synthetic graft, with restoration of the normal borders of the acetabular cavity, including the roof, floor, anterior column, and posterior column (indicated by arrows).

X-rays were taken at various intervals after the surgery (one, three, six, and 12 months) and revealed positive changes in the patient's condition. These included a decrease in lesion size, good incorporation of grafts, and thickening of the lesion walls (Figure 4).

## Discussion

Performing the surgery for ABC during the Syrian conflict posed additional challenges due to the limited healthcare resources. However, despite the challenging circumstances, our team was able to achieve excellent results. Acetabular ABCs of the acetabulum present challenging therapeutic dilemmas, with potential complications such as pathological fracture, protrusio acetabuli, and hip subluxation/dislocation. The weight-bearing line passes through the posterior angle of the dome of the acetabulum, where our patient's lesion was located, increasing the risk of pathological fracture. Surgery was necessary to prevent this outcome.

In previous studies, the treatment of ABCs originating from pelvic bones has been acknowledged as a formidable challenge, particularly for experienced orthopedic oncologists. Upon confirming the diagnosis, meticulous pre-operative planning is deemed essential to identifying the optimal therapeutic approach. Open surgical methods have consistently emerged as the most effective means to eradicate the disease. For pelvic ABCs in children and adolescents, precise curettage and bone grafting have been recognized as the best therapeutic choices [8-11].

In the study conducted by Novais et al. [12], they shared their insights on 13 pediatric aneurysmal bone cysts (ABCs) affecting the pelvis, revealing a minimal local recurrence rate of 7.7%, with only one recurrence among the 13 cases. The study revealed that curettage with bone grafting exhibited a significantly lower recurrence and complication rate. During our patient's operation, the curettage of the medial wall of the acetabulum posed the most difficulty, requiring fine instruments.

The recurrence of ABCs often occurs within 24 months after a primary intervention [9]. In our case, the large extension of the tumor and difficult accessibility, along with medial wall perforation, increased the risk of recurrence and hip joint destruction. Close monitoring and follow-up are necessary to ensure the patient's continued recovery, particularly given the challenging circumstances of the Syrian conflict. The patient was followed closely for two years, and the patient made a full recovery. It is considered a great achievement in light of the ongoing conflict in Syria.

## Conclusions

Performing surgery for acetabular ABCs during the Syrian conflict presented significant challenges due to the limited healthcare resources. Nevertheless, our team was able to achieve excellent results by employing the curettage with bone grafting method, which proved to be effective in preventing recurrence. The ABCs location increased the risk of a pathological fracture, making surgery necessary to avoid this outcome. Despite the high risk of recurrence, the patient made a full recovery, which is considered a great achievement in light of the ongoing conflict in Syria. Close monitoring and follow-up are essential to ensuring the patient's continued recovery. Overall, this case highlights the importance of adapting to challenging situations and using effective treatment methods to achieve positive outcomes.

## References

[1] Goyal A, Tyagi I, Syal R, Agrawal T, Jain M (2006). Primary aneurysmal bone cyst of coronoid process. BMC Ear Nose Throat Disord.

[2] Azar FM, Canale ST, Beaty JH (2021). Campbell's Operative Orthopaedics, 4-Volume Set, 14th Edition. J. B. Frederick Azar, Campbell’s Operative Orthopaedics, 14th Edition., 4 vol.

[3] Gadre KS, Zubairy RA (2000). Aneurysmal bone cyst of the mandibular condyle: report of a case. J Oral Maxillofac Surg.

[4] Pelo S, Gasparini G, Boniello R, Moro A, Amoroso PF (2009). Aneurysmal bone cyst located in the mandibular condyle. Head Face Med.

[5] Calleja JM, Carretero JL, Martín JG, Burgueño M (2007). Aneurysmal bone cyst of the mandible: case presentation and review of the literature. Med Oral Patol Oral Cir Bucal.

[6] Qandeel M, Sommer J (2022). Syria conflict and its impact: a legal and environmental perspective. Journal of International Humanitarian Legal Studies.

[7] Polkowski GG, Clohisy JC (2010). Hip biomechanics. Sports Med Arthrosc Rev.

[8] Dawod MS, Alisi MS, Rabab'a H, Abdulelah AA, Almaaitah HW, Haddad B (2022). Surgical management of aneurysmal bone cyst of the pubis: a case report and review of literature. Int Med Case Rep J.

[9] Restrepo R, Zahrah D, Pelaez L, Temple HT, Murakami JW (2022). Update on aneurysmal bone cyst: pathophysiology, histology, imaging and treatment. Pediatr Radiol.

[10] Strohm JA, Strohm PC, Kühle J, Schmal H, Zwingmann J (2022). Management of juvenile and aneurysmal bone cysts: a systematic literature review with meta-analysis. Eur J Trauma Emerg Surg.

[11] Deventer N, Deventer N, Gosheger G, de Vaal M, Vogt B, Budny T (2021). Current strategies for the treatment of solitary and aneurysmal bone cysts: a review of the literature. J Bone Oncol.

[12] Novais EN, Zimmerman AK, Lewallen LW, Rose PS, Sim FH, McIntosh AL (2014). Functional outcomes and quality of life following surgical treatment of aneurysmal bone cysts of the pelvis in children. J Child Orthop.

